# Population pharmacokinetic analyses for telavancin using data from healthy subjects and patients with infections

**DOI:** 10.1128/aac.01382-24

**Published:** 2025-06-12

**Authors:** Scott A. Van Wart, M. Courtney Safir, Sujata M. Bhavnani, Thomas P. Lodise, Christopher M. Rubino

**Affiliations:** 1 Institute for Clinical Pharmacodynamics 537914, Schenectady, New York, USA; 2 Department of Pharmacy Practice, Albany College of Pharmacy and Health Sciences 1091, Albany, New York, USA; Providence Portland Medical Center, Portland, Oregon, USA

**Keywords:** telavancin, population pharmacokinetics, patients, healthy subjects

## Abstract

Telavancin is an intravenously administered lipoglycopeptide antibiotic active against clinically relevant gram-positive pathogens. In these analyses, a population pharmacokinetic (PK) model was constructed to describe the time course of telavancin in plasma and epithelial lining fluid (ELF) using data from healthy subjects and patients with complicated skin and skin-structure infections, hospital-acquired and ventilator-associated bacterial pneumonia, or uncomplicated bacteremia across Phases 1–4 of clinical development. Data from 1,205 individuals pooled from 21 studies contributed a total of 9,088 telavancin plasma concentrations. The final model for telavancin was a two-compartment model with zero-order intravenous input and linear elimination. Dialysis clearance was included as part of the base structural PK model; the relationship between telavancin clearance and creatinine clearance was included *a priori*. Body weight, age, and infection type were identified as statistically significant predictors of the interindividual variability (IIV) in total clearance. Body weight, age, and infection type were also identified as statistically significant predictors of IIV for the central and peripheral volumes of distribution. Only body weight was found to be a significant predictor of the IIV in distributional clearance. The model for ELF did not reveal any appreciable biases and determined the average free-drug ELF penetration ratio to be 73.0%. In summary, the population PK model characterized the time course of telavancin in both plasma and ELF robustly, captured the impact of clinically meaningful patient covariate effects, including removal of drug due to hemodialysis, and provided reliable individual *post hoc* estimates of exposure in subjects enrolled in the clinical studies.

## INTRODUCTION

Infections due to gram-positive pathogens can cause serious illnesses such as complicated skin and skin structure infections (cSSSI), hospital-acquired and ventilator-associated bacterial pneumonia (HABP and VABP, respectively), infective endocarditis, and bacteremia. Telavancin is an intravenously (IV) administered lipoglycopeptide antibiotic that is active against clinically relevant gram-positive pathogens, including methicillin-resistant *Staphylococcus aureus* (1). Telavancin is approved in the United States, Saudi Arabia, and Russia for the treatment of adults with cSSSI, as well as HABP and VABP (1–3).

Previously, population pharmacokinetic (PK) models ([4, 5], data on file, Cumberland Pharmaceuticals, Inc.) had been constructed using data from telavancin-treated subjects or patients from clinical studies and were used to estimate drug exposures, as well as conduct pharmacokinetic-pharmacodynamic (PK-PD) target attainment analyses ([6], data on file, Cumberland Pharmaceuticals, Inc.). Since these previous analyses were conducted, additional telavancin studies have been completed. These additional studies included several Phase 1 studies in healthy subjects ([7, 8], data on file, Cumberland Therapeutics, Inc.), including a study in obese subjects (9), a study in patients with *S. aureus* bacteremia (10), and a study in patients receiving hemodialysis (11).

The objectives for these analyses were to use the data from these additional studies, along with the previously evaluated data, to develop a more comprehensive population PK model that describes the time course of telavancin in plasma, including removal of drug due to hemodialysis; to identify subject covariates that significantly impact the variability in telavancin PK; and to derive an expanded population PK model to characterize the time course of telavancin in plasma and epithelial lining fluid (ELF).

## RESULTS

### Data

The final analysis data set contained 1,205 subjects and 9,088 telavancin plasma concentrations following single and multiple doses of telavancin across 21 studies. A brief description of each study is provided in Table S1. Plasma PK samples were collected up to 96 hours after a dose. A summary of the number of subjects and samples included in the population PK analyses, after excluding data below the lower limit of quantitation (LLOQ) along with any data excluded for other reasons, is provided based on the full PK data set (i.e., PK analysis population) and by study in Table S2. Summary statistics of baseline subject descriptors for the PK analysis population are presented in Table 1.

**TABLE 1:** Summary statistics of subject demographics, clinical laboratory measures, and disease-related indices for the PK analysis population (*N* = 1,205)^*a*^

Variable	Mean (SD), range or *N* (%)
Age (years)	47.1 (18.4), 18–100
Weight (kg)	79.7 (21.5), 33.6–227
Height (cm)	171 (10.6), 122–203
BSA (m^2^)	1.92 (0.259), 1.22–2.96
BMI (kg/m^2^)	27.3 (6.99), 12.3–88.8
CLcr (mL/min/1.73 m^2^)	83.7 (36.2), 0–203
Race	
Caucasian	916 (76.1)
Black	157 (13.1)
Asian	55 (4.57)
Other	77 (6.39)
Sex	
Male	744 (61.7)
Female	461 (38.3)
Renal function group^*b*^	
Normal	569 (47.3)
Mild impairment	335 (27.8)
Moderate impairment	181 (15.0)
Severe impairment	112 (9.29)
CKD5	8 (0.66)
Infection type	
Healthy	409 (33.9)
HABP/VABP	221 (18.3)
Bacteremia	18 (1.5)
cSSSI	557 (46.2)

^*a*^ BMI, body mass index; BSA, body surface area; CKD5, chronic kidney disease stage 5; *N*, number of subjects or observations; and SD, standard deviation.

^*b*^ Renal function categories are defined as follows: normal, CLcr ≥ 90 mL/min/1.73 m^2^; mild impairment, CLcr ≥ 60 to <90 mL/min/1.73 m^2^; moderate impairment, CLcr ≥ 30 to <60 mL/min/1.73 m^2^; severe impairment, CLcr < 30 mL/min/1.73 m^2^ and not on hemodialysis; and CKD5, on hemodialysis.

### Population pharmacokinetic model development in plasma

**Fig 1:**
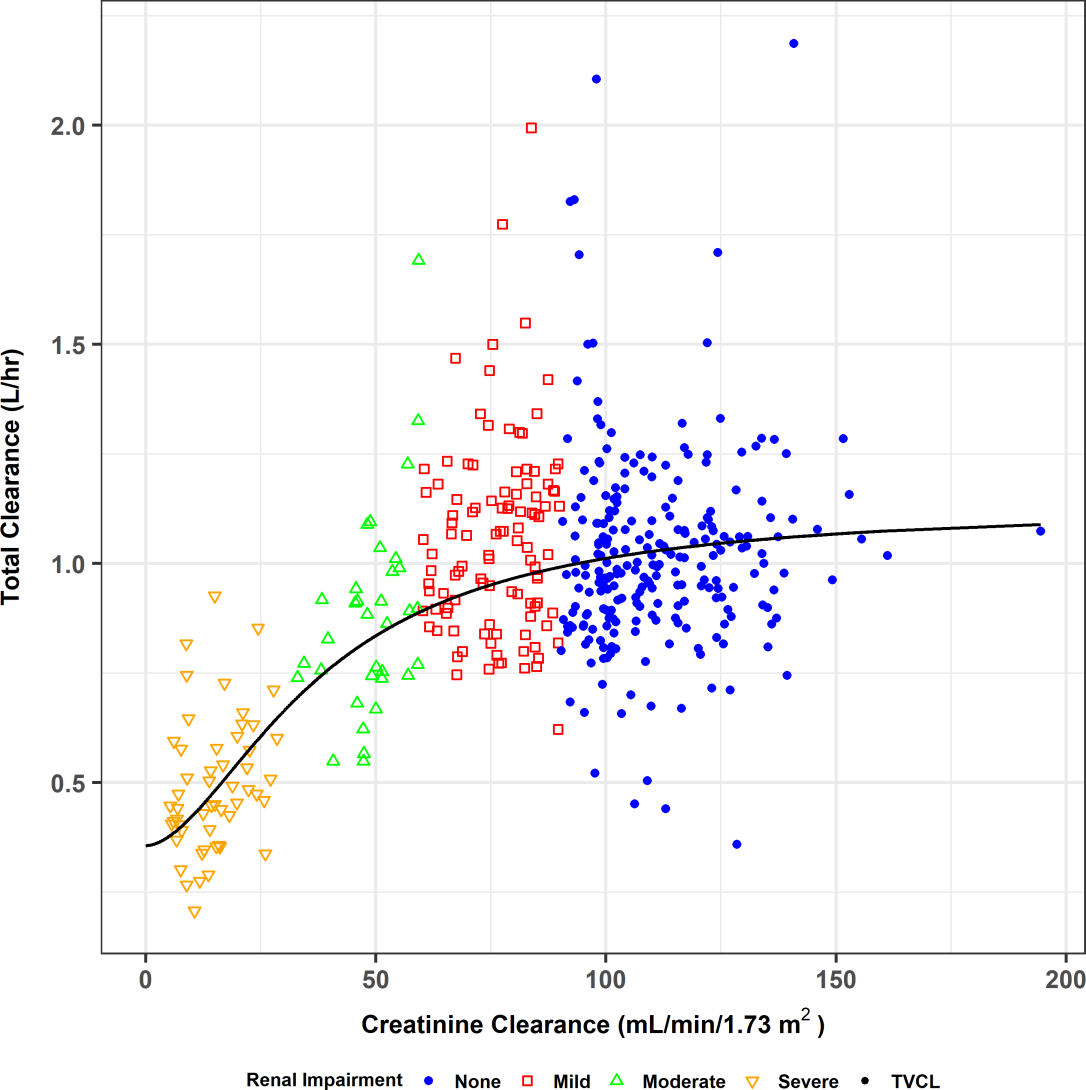
Relationship between total CL and CLcr for the two-compartment structural population PK model fit to the data from healthy subjects (Phase 1 studies).

The adequacy of the base structural PK model was first assessed using only the data from healthy subjects prior to re-fitting the structural PK model to the combined data set from healthy subjects and infected patients and evaluating additional covariate effects. Of the various models attempted, the most parsimonious population PK model fit to the data from healthy subjects enrolled in the Phase 1 studies was a two-compartment model with fixed zero-order IV input and first-order (linear) elimination, with the relationship between renal clearance (CL_R_) and creatinine clearance (CLcr) characterized using a Hill-type function and an intercept to represent non-renal clearance (CL_NR_). Note that the Hill-type function for the relationship between CL_R_ and CLcr was chosen over the alternative functional forms tested within NONMEM (linear and power) as it provided the best apparent fit and allowed the relationship between CL_R_ and CLcr to be relatively flat across the range of normal renal function. This was consistent with the individual *post hoc* fitted parameter estimates. The fitted relationship between total CL and CLcr is provided in Fig. 1 for visualization purposes prior to including the more variable and sparsely sampled PK data from infected patients. All parameters were associated with excellent precision (percent standard error of the population mean estimate [%SEM] < 15%). Goodness-of-fit plots demonstrated excellent agreement between the observed telavancin plasma concentrations and both the population predicted (*r*^2^ = 0.90) and individual *post hoc* predicted (*r*^2^ = 0.98) concentrations.

The two-compartment model used to characterize the PK data from healthy subjects from Phase 1 studies was then fit to all available data from Phase 1 to 4 clinical studies. At this point, several model modifications were required. First, dialysis clearance (CL_DL_) was estimated only during those periods where intermittent hemodialysis (IHD) was active and was fixed to a value of zero when IHD was not operative. An additive increase in the central volume of distribution (Vc) was estimated in the model when PK samples were collected more than 48 hours after the last active IHD session (this parameter was later fixed in the model to the final estimate in order to achieve successful minimization). This was due to the fact that telavancin plasma concentrations were higher when the subject immediately came off IHD and subsequently fell, likely due to fluid depletion during IHD and accumulation between sessions. Interindividual variability (IIV) was estimated for systemic clearance (CL), CL_DL_, Vc, distributional clearance between the central and peripheral compartments (CLd), volume of distribution for the peripheral compartment (Vp), and for some individuals, the duration of infusion (D1) using exponential error models. An additive plus constant coefficient of variation (CCV) error model best described residual variability. Separate CCV error terms were estimated in the model based on the study phase. The base structural PK model included three separate CCV terms to describe residual variability for each phase of clinical development (Phases 1 and 4 were combined into a single value). With the exception of IIV for CL_DL_, all parameters were estimated with acceptable precision. The IIV for CL_DL_ had a higher degree of uncertainty (%SEM = 80.2% in the base structural model), most likely due to the fact that there were only eight subjects in the PK database to inform this parameter.

The stepwise forward selection process identified 13 covariate-parameter relationships that were statistically significant (*α* = 0.01), which were included in the population PK model (Table S3). A scatterplot matrix of IIV terms (ETAs) for the structural PK parameters in the full multivariable model (i.e., the model that resulted from forward selection) revealed strong correlations between CL and Vc and between CLd and Vp. Inclusion of these covariance terms in the full multivariable model resulted in a more statistically significant reduction in the NONMEM minimum value of the objective function (MVOF; 441 units, *P* < 0.00001) than estimating the full omega block; therefore, these two covariance terms were included in the model moving forward. The full multivariable population PK model was then subjected to a stepwise backward elimination procedure in which each covariate effect in the model except CLcr was removed in a univariate fashion and tested for statistical significance (*α* = 0.001). The only covariate-parameter relationship to be removed from the model during backward elimination was that between age and CLd (*P* = 0.05841 upon removal).

**Fig 2:**
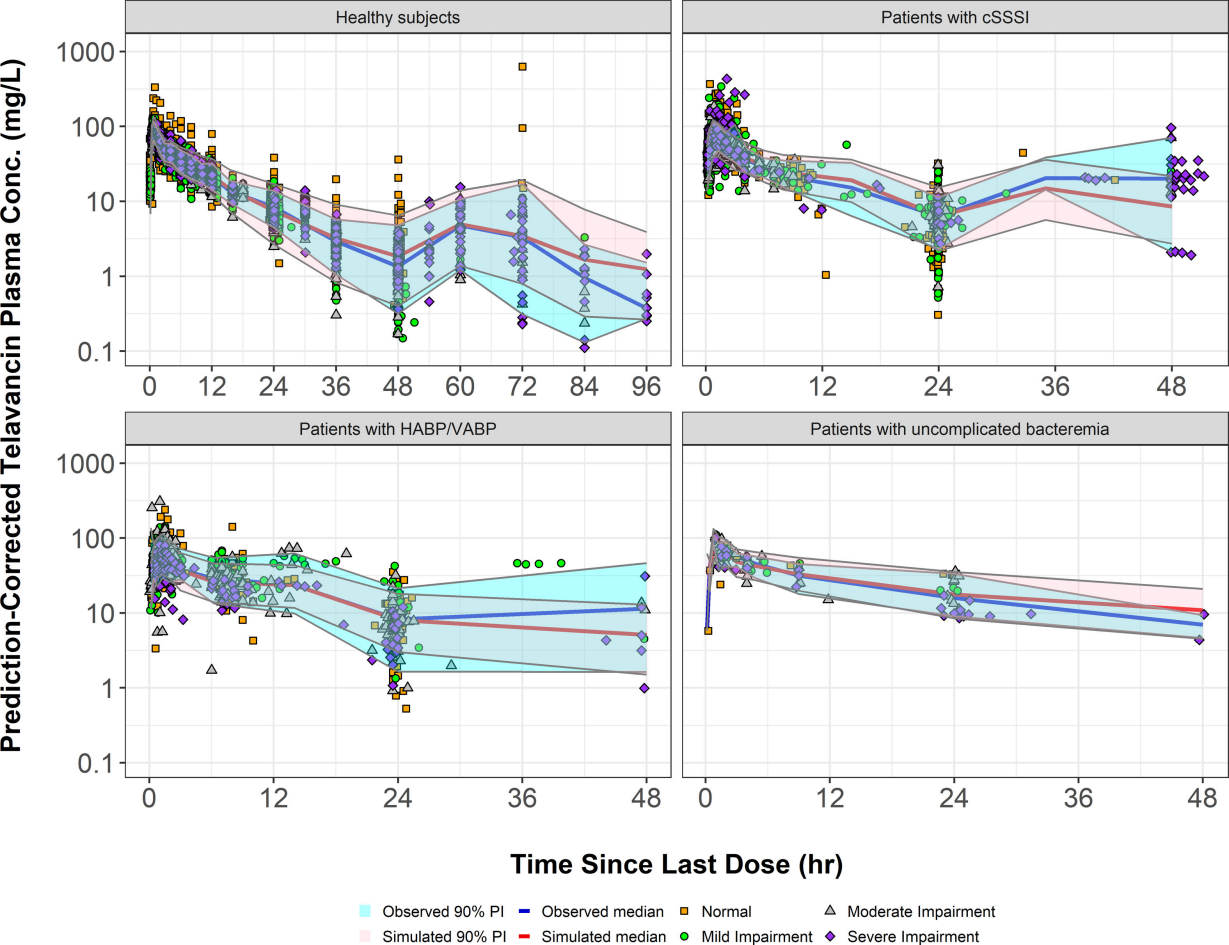
Prediction-corrected visual predictive check for the final population PK model for telavancin in all populations of interest. PI, prediction interval. Renal function categories are defined as follows: normal, CLcr ≥ 90 mL/min/1.73 m^2^; mild impairment, CLcr ≥ 60 to <90 mL/min/1.73 m^2^. moderate impairment, CLcr ≥ 30 to <60 mL/min/1.73 m^2^. severe impairment, CLcr < 30 mL/min/1.73 m^2^ and not on hemodialysis; and CKD5, on hemodialysis.

The final population PK model in both healthy subjects and patients with infections was confirmed to be a two-compartment model with zero-order IV input and first-order elimination, incorporating the previously mentioned adjustments for CL_DL_ and Vc. The final population pharmacokinetic parameter estimates and their associated %SEM based upon the fit of the model to the full data set and subsequent to the bootstrap analysis are provided in Table 2. The goodness-of-fit plots for the final population PK model provided in Fig. S1 show strong agreement between the observed telavancin plasma concentrations and the population predicted concentrations (*r*^2^ = 0.82), as well as the individual *post hoc* predicted concentrations (*r*^2^ = 0.96). Model qualification procedures confirmed the robustness of the final model. The overall distribution of normalized prediction distribution errors (NPDE) appeared to be symmetrical around a value of zero and did not appear to deviate from a normal distribution (data not shown). Prediction-corrected visual predictive check (PC-VPC) plots show reasonable agreement between the 5th, 50th, and 95th percentiles of the observed and the individual simulated telavancin concentrations across time intervals for all populations of interest (Fig. 2). Furthermore, there did not appear to be any bias with respect to renal function in each plot.

**TABLE 2:** Summary statistics of the bootstrap PK parameters in comparison to the final population PK model parameter estimates and associated standard errors^*a*^

Parameter	Final model		Bootstrap statistics (*N* = 200)			
	Final estimate	%SEM	Mean	Median	%CV	90% CI
CL						
CL_NR_ (L/hour)	0.407	2.50	0.399	0.402	2.55	0.374–0.414
Relationship between CL_R_ and CLcr						
CL_R, max_ (L/hour)	1.04	2.59	1.04	1.04	2.22	0.982–1.08
Baseline CLcr_50_ (mL/min/1.73 m^2^)	68.3	3.00	67.2	67.8	3.33	61.9–69.5
Hill coefficient	1.88	3.02	1.87	1.87	2.77	1.76–1.98
CL-TBW power	0.532	6.21	0.516	0.516	8.97	0.422–0.598
CL-age power	0.0921	16.3	0.0926	0.0912	22.0	0.0509–0.133
CL-proportional increase for bacteremia/HABP/VABP patients	0.418	5.80	0.408	0.403	11.1	0.319–0.500
CL-proportional increase for cSSSI patients	0.228	6.92	0.227	0.226	8.98	0.183–0.266
Vc						
Coefficient (L)	5.75	1.24	5.70	5.71	1.27	5.54–5.85
Vc-TBW power	0.469	11.2	0.438	0.442	15.7	0.303–0.584
Vc-age power	0.188	9.09	0.184	0.183	14.0	0.133–0.236
Vc-proportional increase for females	−0.0584	19.5	−0.0583	−0.0577	24.0	−0.0898–−0.0293
Vc-proportional increase for bacteremia/HABP/VABP patients	0.578	7.39	0.600	0.599	10.7	0.494–0.731
Vc-proportional increase for cSSSI patients	0.313	7.20	0.299	0.300	9.66	0.236–0.354
Vc-increase for CKD5 subjects after >48 hours has elapsed between IHD sessions (L)	1.55	25.8	1.57	1.56	30.9	0.514–2.54
CLd						
Coefficient (L/hour)	3.73	1.76	3.78	3.75	2.34	3.64–3.96
Vc-CLd-TBW power	0.772	14.2	0.759	0.764	19.0	0.444–1.05
Vp						
Coefficient (L)	5.52	1.30	5.56	5.55	1.38	5.43–5.74
Vp-TBW power	0.976	7.15	0.971	0.969	8.58	0.824–1.14
Vp-age power	0.272	8.28	0.289	0.289	9.24	0.240–0.346
Vp-BMI power	−0.308	24.0	−0.325	−0.317	20.1	−0.468–−0.212
Vp-proportional increase for bacteremia/HABP/VABP patients	0.329	14.6	0.288	0.295	26.3	0.129–0.423
Vp-proportional increase for cSSSI patients	0.118	23.1	0.127	0.125	19.4	0.0781–0.181
CL_DL_ (L/hour)	1.77	11.1	1.79	1.77	10.3	1.43–2.18
*ω*^2^ for CL	0.0810 (28.5% CV)	3.26	0.0805	0.0806	7.93	0.0688–0.0934
*ω*^2^ for Vc	0.0783 (28.0% CV)	4.41	0.0778	0.0774	12.9	0.0586–0.0967
*ω*^2^ for CLd	0.128 (35.8% CV)	11.1	0.133	0.129	10.5	0.119–0.173
*ω*^2^ for Vp	0.0477 (21.8% CV)	7.69	0.0502	0.0497	13.8	0.0369–0.0634
*ω*^2^ for D1	0.0793 (28.2% CV)	9.10	0.0795	0.0789	4.62	0.0727–0.0874
*ω*^2^ for CL_DL_	0.0651 (25.5% CV)	135	0.0578	0.0619	58.6	0.000944–0.129
Covariance between CL and Vc	0.0622	4.64	0.0619	0.0614	11.9	0.0486–0.0770
Covariance between CLd and Vp	0.0565	9.10	0.0598	0.0592	15.5	0.0419–0.0798
Residual variability (σ^2^)						
Additive component	0.326	3.55	0.341	0.330	13.4	0.277–0.453
CCV component for Phase 1 and 4 studies	0.00984	0.86	0.00987	0.00983	8.61	0.00833–0.0116
CCV component for Phase 2 studies	0.0169	3.25	0.0171	0.0170	13.3	0.0134–0.0228
CCV component for Phase 3 studies	0.0441	2.04	0.0436	0.0439	10.2	0.0347–0.0535

^*a*^ CI, confidence interval; CLcr_50_, creatinine clearance at which CL_R_ is half-maximal; CL_R,max_, maximum renal clearance; %CV, percent coefficient of variation; D1, infusion duration; *ω*^2^, interindividual variability; and TBW, total body weight.

The final population PK model for telavancin provided unbiased and reasonably precise estimates of CL using CLcr (including subjects with severe renal impairment requiring dialysis) along with other demographic and disease-related information. A plot of the individual *post hoc* CL versus the typical value CL (Fig. S2) showed that the bias was minimal (median percent predicted error [PE%] within ±2%), and the precision was within the acceptable range of <30% (median absolute predicted error [|PE%|] was approximately 19%) irrespective of patient population. Therefore, the model was deemed acceptable for the purpose of estimating steady-state area under the concentration-time curve from time 0 to 24 hours (AUC_0-24_) values in those patients who participated in the Phase 2 or 3 clinical studies but who did not have any telavancin plasma PK samples available for analysis.

### Pharmacokinetic analysis of epithelial lining fluid data

**Fig 3:**
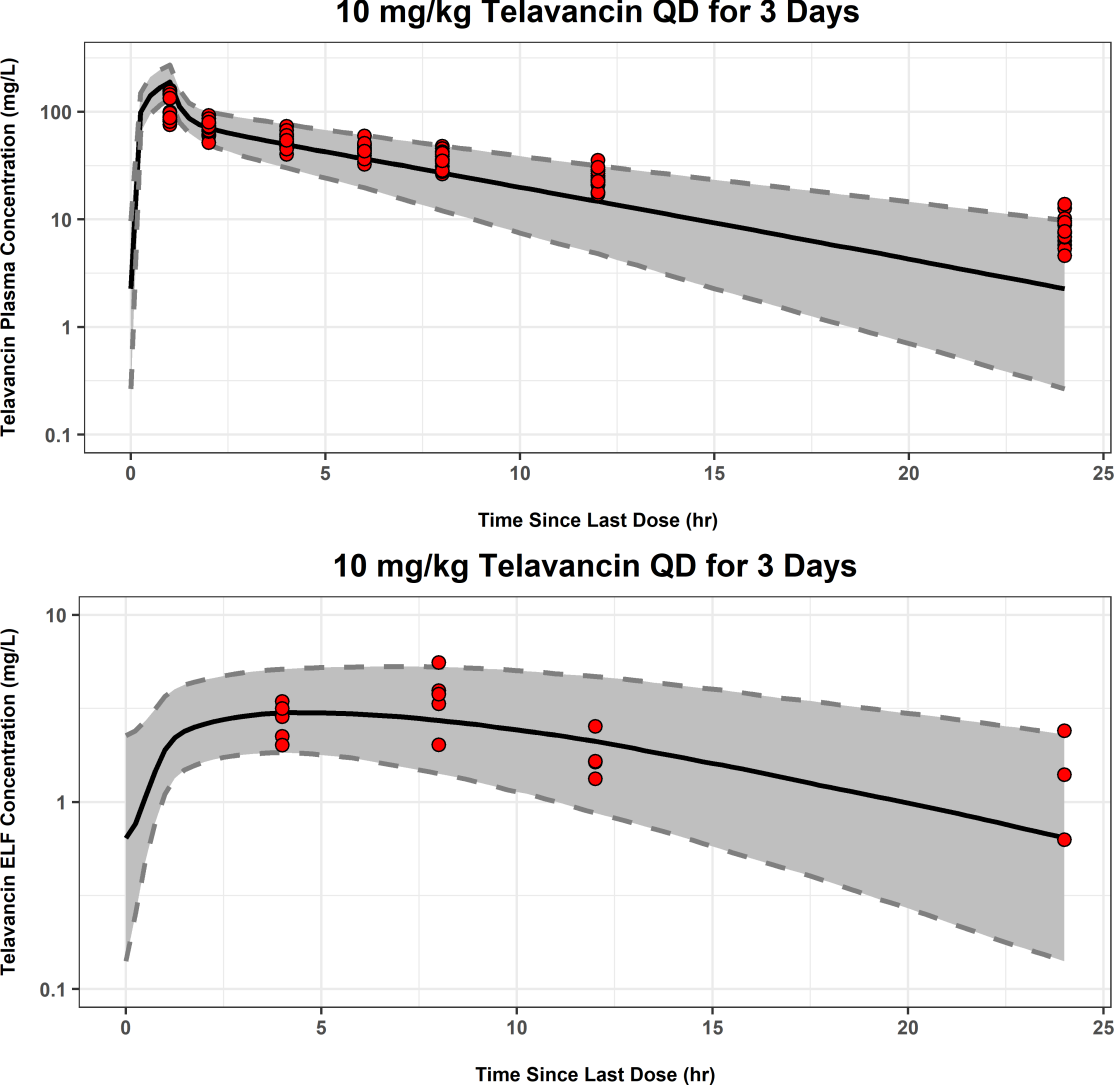
Monte Carlo simulation and visual predictive check for the population PK model fit to the telavancin plasma and ELF data from healthy Phase 1 subjects in Study I6424-108a. For each figure, the red circles are observed concentrations while the solid black line represents the median of the simulated concentrations and black dashed lines represent the 5th and 95th percentiles of the simulated concentrations. The grey shaded regions are the 90% prediction intervals for the 5th and 95th percentiles of the simulated concentrations. QD, once daily. Two subjects had telavancin ELF concentrations that were below the lower limit of quantitation and were thus, excluded from this figure.

A population PK analysis approach was then used to characterize the time course of telavancin in the plasma and ELF upon repeated IV dosing of telavancin 10 mg/kg of body weight to only the 20 healthy subjects who participated in Study I6424-108a. Concentrations from two subjects were below the LLOQ and were thus, excluded from the analyses, resulting in 18 concentrations from 18 subjects. The ELF population PK model parameter estimates are provided in Table S4. By fixing the plasma PK parameters to the individual *post hoc* values, it was possible to predict ELF concentrations using a biophase model with unidirectional first-order transfer from the central compartment to ELF (k_13_) and first-order elimination from the ELF compartment (k_30_). In order to allow for the estimation of IIV in k_30_, since only one ELF measurement was collected per subject, the residual variability of ELF data was fixed to be equal to that previously estimated for the plasma PK data in healthy subjects from Phases 1 and 4 studies. Examination of goodness-of-fit plots for the ELF PK data from Study I6424-108a did not reveal any appreciable biases (Fig. S3). A simulation-based diagnostic procedure, using the mean and standard deviation (SD) of observed demographics and lab covariates from these same healthy subjects, was subsequently performed and overlaid upon the observed plasma and sparse sampling ELF data from the healthy subjects in Study I6424-108a. In this simulation, the parameter estimates from the modified final population PK model were utilized along with the mean (standard deviation) of the subject covariate effects in the model, such as age and body weight from healthy subjects in Study I6424-108a. In general, this visual predictive check (shown in Fig. 3) demonstrated that the model reasonably predicted the observed plasma and ELF concentration-time data.

Summary statistics of telavancin steady-state AUC_0-24_ in plasma and ELF, as well as the ELF penetration ratios calculated using total-drug and free-drug plasma AUC_0-24_, are presented in Table 3 for the healthy subjects who participated in Study I6424-108a. Based on free-drug plasma concentrations of telavancin, the average free-drug ELF penetration ratio was determined to be 73.0%.

**TABLE 3:** Summary statistics of telavancin steady-state AUC_0-24_ in plasma and ELF, as well as the ELF penetration ratios calculated using total-drug and free-drug plasma AUC_0-24_ for healthy subjects in Study I6424-108a^*a*^

Variable	*N*	Mean (SD)	Median	Minimum	Maximum
Plasma AUC_0-24_ (mg·hour/L)	20	750 (103)	757	570	954
ELF AUC_0-24_ (mg·hour/L)	20	54.5 (16.1)	50.6	38.3	99.0
ELF penetration ratio (total drug)^*b*^	20	0.0730 (0.0204)	0.0683	0.0506	0.139
ELF penetration ratio (free drug)^*c*^^,^^*d*^	20	0.730 (0.204)	0.683	0.506	1.39

^*a*^ *N*, number of subjects or observations.

^*b*^ Calculated as ELF AUC_0-24_/total-drug plasma AUC_0-24_.

^*c*^ Calculated as ELF AUC_0-24_/(0.1 total-drug plasma AUC_0-24_).

^*d*^ Based upon using a protein binding estimate of 90% in humans (1).

## DISCUSSION

The primary goal of these analyses was to develop a population PK model to describe the time course of telavancin in plasma using all available data from healthy subjects and patients with cSSSI, HABP, VABP, and uncomplicated bacteremia across Phases 1–4 of clinical development and in subjects covering the entire spectrum of renal function. These analyses allowed for the use of PK data from several studies not included in previous population PK analyses ([5], data on file, Cumberland Pharmaceuticals, Inc.), including Phase 2 patients with *S. aureus* bacteremia (10) and special populations, such as subjects with obesity and chronic kidney disease stage 5 (CKD5) subjects undergoing IHD (9, 11). In addition to considering these data, actual patient dosing histories in the Phase 2 or 3 clinical studies were used, and the covariate analyses included the evaluation of CLcr as a time-varying covariate. A formal covariate analysis was also performed to help identify which other demographic, clinical laboratory, and disease-related covariates explained IIV in telavancin PK.

The final population PK model for telavancin, which best described the healthy subject and infected patient data, was a two-compartment model with a zero-order rate constant for the IV infusion and first-order elimination, which is consistent with the results of the previous analyses (5). The relationship between CL_R_ and CLcr was described using a sigmoidal Hill-type function; the total CL of telavancin included an intercept to represent CL_NR_. To account for IHD, CL_DL_ was included in the model; this parameter describes the extent of increased drug clearance during dialysis and was estimated during periods of IHD and fixed to a value of zero when IHD was not operative. An increase in Vc was also estimated when PK samples were collected more than 48 hours after the last active IHD session in order to account for potential excess fluid accumulation. Incorporating these relationships ensured that the population PK model was built upon sufficient clinical data across the full spectrum of renal function and had the necessary flexibility to reliably perform simulations to assess the impact of renal impairment and IHD on telavancin exposure.

Creatinine clearance had been identified as a clinically significant covariate effect for telavancin CL in previous population PK analyses ([5], data on file, Cumberland Therapeutics, Inc.). However, given that baseline CLcr was used to inform the CL-CLcr relationship and that almost all of the PK data were collected from infected patients after at least 3 days of therapy with telavancin, the effect of CLcr over time on CL was considered. The use of CLcr as a time-varying covariate effect provided a more reliable characterization of the CL-CLcr relationship and also enabled projection of changes in telavancin exposures during therapy.

The formal covariate evaluation identified body weight, age, and infection type as statistically significant predictors of CL. Body weight, age, sex (Vc only), body mass index (BMI) (Vp only), and infection type were statistically significant predictors of both Vc and Vp. Body weight was the only statistically significant predictor of CLd. These additional covariate effects collectively reduced the magnitude of the unexplained IIV (percent coefficient of variation [%CV]) from 31.7% to 28.4% for CL, from 42.6% to 28.4% for Vc, from 38.5% to 36.1% for CLd, and from 26.0 to 22.9% for Vp in the final population PK model relative to the base structural PK model. The fact that body weight and age were statistically significant covariate effects on CL even after accounting for CLcr in the model (considering CLcr was further normalized to a body surface area [BSA] of 1.73 m^2^ to adjust for the confounding effect of body size on serum creatinine production) may be due to the fact that telavancin is partially eliminated via metabolism in addition to renal clearance (1, 7). Importantly, the addition of body weight, age, and infection type to the model for telavancin CL had a modest impact on the unexplained variability (i.e., the IIV in CL was 31.7% in the base structural PK model applied to the pooled Phase 1, 2, and 3 data set and decreased to only 28.5% in the final model). Of note, both body weight and BMI were found to be predictive of the IIV in telavancin Vp. Given that BMI is a measure of relative obesity, the inclusion of both measures is likely reflective of the impact of both body size and obesity on telavancin PK. In a separate analysis of telavancin in obese subjects (9), body weight did not appear to have an effect on CL in healthy subjects, although the volume of distribution at steady state did tend to increase with body weight. Consequently, that analysis suggested that fixed dosing in obese patients with normal renal function may be appropriate. As the results presented herein do not reach the same conclusion, further analysis of the effects of body size on telavancin PK may be warranted.

The final population PK model was successfully qualified by conducting a PC-VPC, and the robustness of the model due to perturbations of the data was established by completing a non-parametric bootstrap exercise. In addition, the final population PK model for telavancin appeared to have the ability to provide unbiased and reasonably precise estimates of CL using demographic and disease-related information, which is useful for predicting steady-state AUC_0-24_ values in patients for whom telavancin plasma PK data are not available.

Herein, we found the mean free-drug ELF penetration ratio of telavancin to be 73%, which is consistent with the median penetration ratio of 73% reported by Lodise et al. (4). The mean penetration ratio determined by Lodise et al. was 101%, but it was noted that outliers may have had a significant effect on mean values. Compared to oritavancin, another glycopeptide antibiotic with a reported penetration ratio of 459% (12), this penetration ratio is relatively low. However, the ELF penetration ratio of telavancin is higher than that of dalbavancin and vancomycin, two other glycopeptides. Dalbavancin has a reported ELF penetration of 36% (13), while ELF penetration for vancomycin has been reported to be 41%–56% in healthy volunteers with outliers removed (12, 14).

Despite the robust data set used as the basis for model development, the primary limitations of these analyses center around the following two aspects for which data were limited: (i) the impact of IHD on telavancin PK, and (ii) ELF penetration. As evidenced by the relative imprecision of the IIV on CL_DL_ (135% in the final model fit and 58.6% CV in the bootstrap), the dependence on data from only eight subjects with IHD impeded our ability to estimate the amount of variability in this parameter. Additional data would be needed to fully inform the variability in the impact of IHD on telavancin PK. The sub-model that describes the penetration of telavancin into ELF is informed by data from 20 healthy subjects, each of whom contributed only one bronchoalveolar lavage fluid sample for the determination of telavancin ELF concentration. While this is consistent with what has historically been available for other antibiotics, it nonetheless resulted in an inability to fully inform the inherent variability in telavancin ELF penetration.

Overall, the population PK model presented herein was robust and provided unbiased and precise estimations of telavancin exposures across various patient populations. The model is considered reliable for generating individual *post hoc* estimates of exposure in patients enrolled in the clinical studies, as well as for conducting model-based simulations. Accordingly, this model will allow for future PK-PD analyses for telavancin and simulations to evaluate telavancin dosing regimens in various populations to be conducted.

## MATERIALS AND METHODS

### Data

A total of 21 studies were included in the population PK analysis data set. Twelve of these were Phase 1 studies conducted in healthy subjects ([7, 8, 15–20], data on file, Cumberland Therapeutics, Inc.), one was a Phase 1 study in obese subjects (9), four were Phase 2 or 3 studies in patients with cSSSI (21–23), one was a Phase 2 study in patients with uncomplicated *S. aureus* bacteremia (10), two were Phase 3 studies in patients with HABP or VABP (24), and one was a Phase 4 study in otherwise healthy subjects with renal impairment (11). Of the 21 studies, 4 of the Phase 1 studies, the Phase 2 study in patients with uncomplicated *S. aureus* bacteremia, and the Phase 4 study had not been included in previous population PK analyses ([7–11], data on file, Cumberland Therapeutics, Inc.). The analysis population was defined as all subjects or patients who received at least one dose of telavancin and who had at least one quantifiable telavancin concentration available for analysis. A brief description of all studies included in the current analysis is included in Table S1.

### Data handling

Actual dosing (i.e., infusion start and stop) and PK sampling times were used for the analysis. However, the infusion duration (D1) was fixed to a value of 1 hour for subjects in the Phase 2 or 3 clinical studies in which this information was not recorded. PK sample data were not utilized if date and/or time information were not available. Telavancin concentrations were determined using a liquid chromatography-tandem mass spectrometry assay with a LLOQ of 0.250 µg/mL for all but one Phase 1 study, Study TLV-2015-011, and the Phase 4 study, Study TLV-2014-020, which each had a LLOQ of 0.100 µg/mL ([7–9, 11, 15–20], data on file, Cumberland Pharmaceuticals, Inc.). In the Phase 1 study, Study I6424-108a, the LLOQ in the BAL supernatant for telavancin was 0.02 μg/mL [19]. Data below the LLOQ were excluded from the analysis. Outlier detection was based primarily on visual inspection of individual and pooled plasma and ELF concentration-time data for telavancin. Additional outliers were identified by graphical exploration of individual and population conditional weighted residuals during structural PK model development.

### Population pharmacokinetic model development in plasma

The population PK data set was constructed using SAS version 9.4 (25). All population PK analyses at the time were performed using NONMEM version 7.2 (26) using the first-order conditional estimation method with interaction. To compare hierarchical models, change in the MVOF was evaluated after the addition or deletion of a single fixed or random effects parameter to the structural models. The addition or deletion was considered statistically significant if it contributed to a change of 10.83 units (*P* < 0.001, 1 degree of freedom) in the MVOF for nested models. Failure to achieve successful model minimization or completion of the covariance step may have resulted in opting against selection of that particular structural population PK model despite a statistically significant reduction in MVOF. Population PK models were also assessed using precision in individual and population mean parameter estimation, goodness-of-fit and residual plots, reduction in both interindividual and residual variability, and comparison of Akaike’s Information Criterion for non-nested models.

Previous analyses demonstrated that a two-compartment model with zero-order IV input (*k*_*0*_) and linear first-order elimination best described the time course of telavancin in plasma ([5], data on file, Cumberland Pharmaceuticals, Inc.). Therefore, the same base structural PK model was initially evaluated in the current analysis. The following parameters were fit for a two-compartment model: telavancin CL, Vc, the volume of distribution for the first and second peripheral compartments (Vp1 and Vp2, respectively), and the distribution clearances between the central compartment and either the first or second peripheral compartments (CLd1 and CLd2, respectively). Due to the predominant renal elimination for the total telavancin CL, CL_NR_ and various functions describing the relationship between CL_R_ and CLcr were estimated in the population PK model *a priori*. For comparison purposes, three-compartment models were also evaluated. Interindividual variability (IIV or *ω*^2^) was modeled for each PK parameter using exponential error models, where appropriate. Different error models (i.e., additive, proportional, and combined additive plus constant CCV error models) were tested to describe residual variability (*σ*^2^).

The fit of the base structural PK model was first assessed using the data only from healthy subjects prior to re-fitting the model to the combined data set from healthy subjects and infected patients and performing the covariate analysis. To account for IHD, CL_DL_ was added to the model and estimated during time periods when IHD was active and set to zero during time periods when IHD was inactive.

After establishing the base structural PK model, covariate analyses were conducted using NONMEM. The following continuous covariates were investigated for their ability to explain portions of the IIV in the population PK parameters: age in years, weight in kg, height in cm, BSA in m^2^, BMI in kg/m^2^, and CLcr in mL/min/1.73 m^2^. Creatinine clearance, which was calculated using ideal body weight (total body weight for subjects with total body weight less than ideal body weight) and then normalized to BSA, was employed as a time-varying covariate in the recognition of the fact that renal function may be altered during the course of telavancin treatment. Linear interpolation was used to predict a serum creatinine (Scr) value to provide a more gradual change in the calculated CLcr in between actual measured Scr values. If continuous covariate data were not available for a given individual, the median value of the covariate for the available data was assigned to that individual. Categorical covariates that were investigated included sex, race, and infection type (cSSSI infection, HABP/VABP infection, or uncomplicated bacteremia infection, and none for healthy subjects).

Covariate analysis was performed using stepwise forward selection (*α* = 0.01) followed by backward elimination (*α* = 0.001). Covariances between the IIV terms were evaluated and estimated in the variance-covariance matrix for the final population PK model if warranted. The final population PK model for plasma was evaluated using several model qualification procedures, including assessment of the NPDE, construction of PC-VPC plots, and performance of a non-parametric bootstrap evaluation (27).

### Population pharmacokinetic modeling in epithelial lining fluid

After finalization of the population PK model for plasma, a sub-model PK model describing the time course of telavancin in the ELF was constructed based upon urea-corrected (28) bronchoalveolar lavage fluid data (one sample per subject at either 4, 8, 12, or 24 hours post-dose) obtained from 20 healthy adult subjects who participated in Study I6424-108a (19) . Various structural models were evaluated, including a unidirectional biophase model, to characterize the appearance and elimination of telavancin in the ELF. The same population PK model evaluation criteria outlined previously were utilized, where applicable, to assess the fit to the telavancin ELF data.

## SUPPLEMENTAL MATERIAL

Supplemental material aac.01382-24-s0001.pdf Tables S1 to S4; Fig. S1 to S3.

ASM does not own the copyrights to Supplemental Material that may be linked to, or accessed through, an article. The authors have granted ASM a non-exclusive, world-wide license to publish the Supplemental Material files. Please contact the corresponding author directly for reuse.

## References

[1] Vibativ (telavancin) [package insert]. 2023. Cumberland Pharmaceuticals Inc, Nashville, TN.

[2] Tabuk and Cumberland partner to bring innovative antibiotic with life-saving potential to Middle East [press release]. 2022. Cumberland Pharmaceuticals, Nashville, TN. Available from: https://investor.cumberlandpharma.com/news-releases/news-release-details/tabuk-and-cumberland-partner-bring-innovative-antibiotic-life. Retrieved 14 Mar 2025.

[3] Theravance biopharma announces marketing authorization for VIBATIV (Telavancin) in Russia for treatment of multiple infections caused by Gram-positive bacteria, including MRSA [press release]. 2015. Theravance Biopharma, Dublin.

[4] Lodise TP, Gotfried M, Barriere SL, Drusano GL. 2008. Telavancin penetration into human epithelial lining fluid determined by population pharmacokinetic modeling and Monte Carlo simulation. Antimicrob Agents Chemother 52:2300–2304. doi:10.1128/AAC.01110-07 18426898 PMC2443895

[5] Samara E, Shaw JP, Barriere SL, Wong SL, Worboys P. 2012. Population pharmacokinetics of telavancin in healthy subjects and patients with infections. Antimicrob Agents Chemother 56:2067–2073. doi:10.1128/AAC.05915-11 22252798 PMC3318396

[6] Lodise TP, Butterfield JM, Hegde SS, Samara E, Barriere SL. 2012. Telavancin pharmacokinetics and pharmacodynamics in patients with complicated skin and skin structure infections and various degrees of renal function. Antimicrob Agents Chemother 56:2062–2066. doi:10.1128/AAC.00383-11 22252799 PMC3318336

[7] Wong SL, Goldberg MR, Ballow CH, Kitt MM, Barriere SL. 2010. Effect of telavancin on the pharmacokinetics of the cytochrome P450 3A probe substrate midazolam: a randomized, double-blind, crossover study in healthy subjects. Pharmacotherapy 30:136–143. doi:10.1592/phco.30.2.136 20099988

[8] Wong SL, Sörgel F, Kinzig M, Goldberg MR, Kitt MM, Barriere SL. 2009. Lack of pharmacokinetic drug interactions following concomitant administration of telavancin with aztreonam or piperacillin/tazobactam in healthy participants. J Clin Pharmacol 49:816–823. doi:10.1177/0091270009337133 19443680

[9] Bunnell KL, Pai MP, Sikka M, Bleasdale SC, Wenzler E, Danziger LH, Rodvold KA. 2018. Pharmacokinetics of telavancin at fixed doses in normal body weight and obese (Classes I, II, and III) adult subjects. Antimicrob Agents Chemother 62:e02475-17. doi:10.1128/AAC.02475-17 29311094 PMC5913990

[10] Stryjewski ME, Lentnek A, O’Riordan W, Pullman J, Tambyah PA, Miró JM, Fowler Jr VG, Barriere SL, Kitt MM, Corey GR. 2014. A randomized Phase 2 trial of telavancin versus standard therapy in patients with uncomplicated Staphylococcus aureus bacteremia: the ASSURE study. BMC Infect Dis 14:289. doi:10.1186/1471-2334-14-289 24884578 PMC4048626

[11] Gharibian KN, Lewis SJ, Heung M, Segal JH, Salama NN, Mueller BA. 2021. Telavancin pharmacokinetics in patients with chronic kidney disease receiving haemodialysis. J Antimicrob Chemother 77:174–180. doi:10.1093/jac/dkab370 34613416

[12] Rodvold KA, Gotfried MH, Loutit JS, Porter SB. 2004. Plasma and intrapulmonary concentrations of oritavancin and vancomycin in normal healthy adults. Abstract NO. O-254. 14th European Congress of Clinical Microbiology and Infectious Diseases; May 1 to 4, 2004. Prague, Czech Republic. Clin Microbiol Infect. Vol. 10 Supplement 3:44.

[13] Rappo U, Dunne MW, Puttagunta S, Baldassarre JS, Su S, Desai-Krieger D, Inoue M. 2019. Epithelial lining fluid and plasma concentrations of dalbavancin in healthy adults after a single 1,500-milligram infusion. Antimicrob Agents Chemother 63:e01024-19. doi:10.1128/AAC.01024-19 31501147 PMC6811436

[14] Lodise TP, Drusano GL, Butterfield JM, Scoville J, Gotfried M, Rodvold KA. 2011. Penetration of vancomycin into epithelial lining fluid in healthy volunteers. Antimicrob Agents Chemother 55:5507–5511. doi:10.1128/AAC.00712-11 21911567 PMC3232765

[15] Shaw JP, Seroogy J, Kaniga K, Higgins DL, Kitt M, Barriere S. 2005. Pharmacokinetics, serum inhibitory and bactericidal activity, and safety of telavancin in healthy subjects. Antimicrob Agents Chemother 49:195–201. doi:10.1128/AAC.49.1.195-201.2005 15616296 PMC538848

[16] Wong SL, Barriere SL, Kitt MM, Goldberg MR. 2008. Multiple-dose pharmacokinetics of intravenous telavancin in healthy male and female subjects. J Antimicrob Chemother 62:780–783. doi:10.1093/jac/dkn273 18586659

[17] Goldberg MR, Wong SL, Shaw JP, Kitt MM, Barriere SL. 2010. Single-dose pharmacokinetics and tolerability of telavancin in elderly men and women. Pharmacotherapy 30:806–811. doi:10.1592/phco.30.8.806 20653356

[18] Sun HK, Duchin K, Nightingale CH, Shaw JP, Seroogy J, Nicolau DP. 2006. Tissue penetration of telavancin after intravenous administration in healthy subjects. Antimicrob Agents Chemother 50:788–790. doi:10.1128/AAC.50.2.788-790.2006 16436747 PMC1366897

[19] Gotfried MH, Shaw JP, Benton BM, Krause KM, Goldberg MR, Kitt MM, Barriere SL. 2008. Intrapulmonary distribution of intravenous telavancin in healthy subjects and effect of pulmonary surfactant on in vitro activities of telavancin and other antibiotics. Antimicrob Agents Chemother 52:92–97. doi:10.1128/AAC.00875-07 17923490 PMC2223919

[20] Goldberg MR, Wong SL, Shaw JP, Kitt MM, Barriere SL. 2010. Lack of effect of moderate hepatic impairment on the pharmacokinetics of telavancin. Pharmacotherapy 30:35–42. doi:10.1592/phco.30.1.35 20030471

[21] Stryjewski ME, O’Riordan WD, Lau WK, Pien FD, Dunbar LM, Vallee M, Fowler VG, Chu VH, Spencer E, Barriere SL, Kitt MM, Cabell CH, Corey GR, the FAST Investigator Group. 2005. Telavancin versus standard therapy for treatment of complicated skin and soft-tissue infections due to Gram-positive bacteria. Clin Infect Dis 40:1601–1607. doi:10.1086/429914 15889357

[22] Stryjewski ME, Chu VH, O’Riordan WD, Warren BL, Dunbar LM, Young DM, Vallée M, Fowler VG Jr, Morganroth J, Barriere SL, Kitt MM, Corey GR, FAST 2 Investigator Group. 2006. Telavancin versus standard therapy for treatment of complicated skin and skin structure infections caused by gram-positive bacteria: FAST 2 study. Antimicrob Agents Chemother 50:862–867. doi:10.1128/AAC.50.3.862-867.2006 16495243 PMC1426424

[23] Stryjewski ME, Graham DR, Wilson SE, O’Riordan W, Young D, Lentnek A, Ross DP, Fowler VG, Hopkins A, Friedland HD, Barriere SL, Kitt MM, Corey GR, Assessment of Telavancin in Complicated Skin and Skin-Structure Infections Study. 2008. Telavancin versus vancomycin for the treatment of complicated skin and skin-structure infections caused by Gram-positive organisms. Clin Infect Dis 46:1683–1693. doi:10.1086/587896 18444791

[24] Rubinstein E, Lalani T, Corey GR, Kanafani ZA, Nannini EC, Rocha MG, Rahav G, Niederman MS, Kollef MH, Shorr AF, Lee PC, Lentnek AL, Luna CM, Fagon J-Y, Torres A, Kitt MM, Genter FC, Barriere SL, Friedland HD, Stryjewski ME, ATTAIN Study Group. 2011. Telavancin versus vancomycin for hospital-acquired pneumonia due to Gram-positive pathogens. Clin Infect Dis 52:31–40. doi:10.1093/cid/ciq031 21148517 PMC3060890

[25] SAS 9.4 for Windows [computer program]. 2013. SAS Institute Inc, Cary, NC.

[26] Bauer RJ. 2010. NONMEM users guides. NONMEM. Version 7.2. ICON Development Solutions, Ellicott City, MD.

[27] Bergstrand M, Hooker AC, Wallin JE, Karlsson MO. 2011. Prediction-corrected visual predictive checks for diagnosing nonlinear mixed-effects models. AAPS J 13:143–151. doi:10.1208/s12248-011-9255-z 21302010 PMC3085712

[28] Rennard SI, Basset G, Lecossier D, O’Donnell KM, Pinkston P, Martin PG, Crystal RG. 1986. Estimation of volume of epithelial lining fluid recovered by lavage using urea as marker of dilution. J Appl Physiol (1985) 60:532–538. doi:10.1152/jappl.1986.60.2.532 3512509

