# Supplementary material for: Population pharmacokinetic analyses for telavancin using data from healthy subjects and patients with infections
Source: Antimicrob Agents Chemother. 2025 Jun 12;69(7):e01382-24. doi: 10.1128/aac.01382-24 (PMC12217474; doi:10.1128/aac.01382-24)
Supplement: Supplemental material — Tables S1 to S4; Fig. S1 to S3. [file aac.01382-24-s0001.pdf]

Table S1. Summary of clinical studies used to develop the population PK model for telavancin

Study	Phase	Study Design	Subjects/ Patients (n)[a]	Telavancin IV Dosing Regimens	Scheduled PK Sample Collection Times
I6424-101a [1]	1	First-in-man, two-part, randomized, double-blind, placebo-controlled study with ascending doses of telavancin in healthy subjects	54	Part 1: Single ascending doses of 0.25 to 15 mg/kg infused over 0.5, 1, or 2 h<br>Part 2: 7.5, 12.5, or 15 mg/kg q24h for 7 days	Part 1: Up to 24 h post-dose<br>Part 2: Up to 24 h after first dose, and up to 48 h after the last dose
I6424-103a [2]	1	Open-label, single dose study to evaluate telavancin PK in subjects with varying degrees of renal dysfunction	29	Single dose of 7.5 mg/kg infused over 1 h	Day 1: Pre-dose and at 1, 2, 4, 6, 8, 12, 24, 36, and 48 hours post-dose (additional samples drawn at 72 h and 96 h post-dose in subjects with severe renal impairment or on hemodialysis)
I6424-104a [3]	1	Randomized, double-blind, parallel group, sex-stratified, multiple-dose safety and PK study in healthy subjects	80	7.5 or 15 mg/kg doses infused over 1 h q24h for 3 days	Day 3: pre-dose and 0.5, 1, 2, 4, 6, 8, 12, 24, 36, and 48 h post-dose
I6424-105a [4]	1	Open-label, single-dose, sex-stratified study to evaluate the effects of sex on telavancin PK in an elderly population	16	Single dose of 10 mg/kg infused over 1 h	Day 1: 1, 2, 4, 6, 8, 12, 18, 24, 36, and 48 h post-dose
I6424-107a [5]	1	Open-label, single-arm, multiple-dose Phase 1 PK study to evaluate the penetration of telavancin into skin blisters	9	7.5 mg/kg doses infused over 1 h q24h for 3 days	Day 3: Pre-dose, at the end of infusion, and 1, 3, 5, 7, 11, and 23 h after the end of infusion
I6424-108a [6]	1	Open-label, single-arm, multiple-dose Phase 1 PK study assess the penetration of telavancin into pulmonary ELF and alveolar macrophages	20	10 mg/kg doses infused over 1 h q24h for 3 days	Day 3: Pre-dose, at the end of infusion, and 1, 3, 5, 7, 11, and 23 h after the end of infusion<br>BAL: at 4, 8, 12, or 24 hours after the dose on Day 3
0016 [7]	1	Open label, single-dose study to evaluate the effect of hepatic impairment on telavancin PK	16	Single dose of 10 mg/kg infused over 1 h	Day 1: Pre-dose and 1, 2, 4, 6, 8, 12, 24, 36, 48, and 72 h after the start of infusion

Table S1. Summary of clinical studies used to develop the population PK model for telavancin

Study	Phase	Study Design	Subjects/ Patients (n)[a]	Telavancin IV Dosing Regimens	Scheduled PK Sample Collection Times
9809-CL-1407 [2]	1	Open-label, single-dose study to evaluate telavancin PK in subjects with varying degrees of renal function	45	Single dose of 7.5 mg/kg infused over 1 h	Day 1: Pre-dose and 1, 2, 4, 6, 8, 12, 24, 30, 36, and 48 h after the start of infusion. Additional samples at 54, 60, and 72 h after start of infusion for those on hemodialysis.
9809-CL-2403 [2]	1	Open-label, single-dose study to evaluate telavancin PK in subjects with varying degrees of renal function	43	Single dose of 10 mg/kg infused over 1 h	Day 1: Pre-dose and at 0.5, 1, 1.5, 2, 3, 4, 6, 8, 10, 12, 16, 24, 36, 48, 60, 72, 84, and 96 h post-dose
I6424-0032 [8]	1	Double-blind, crossover study to evaluate the potential PK drug-drug interaction between telavancin and midazolam in healthy subjects	16	10 mg/kg dose infused over 1 h q24h for 7 days	Day 7: Pre-dose and 0.25, 0.5, 1, 2, 3, 4, 6, 8, 10, 12, and 24 h after midazolam administration
I6424-0035 [9]	1	Double-blind, crossover study to evaluate the potential PK drug-drug interaction between telavancin and either aztreonam or piperacillin/tazobactam in healthy subjects	26	Single 10 mg/kg dose infused over 1 h	Day 1: Pre-dose, within 1 minute of the end of infusion, and at 0.0833, 0.167, 0.25, 0.333, 0.5, 0.75, 1, 1.5, 2, 3, 4, 6, 8, 12, and 24 h post-dose
9809-CL-3101 [2]	1	Double-blind, placebo-controlled study to assess the PK, safety, and tolerability of single- and multiple-doses of telavancin in healthy Japanese and Caucasian males and females	24	7.5 or 10 mg/kg doses infused over 1 h on Day 1 and q24h on Days 4 to 10	Days 1 and 10: Pre-dose and at 0.5, 1, 1.5, 2, 4, 6, 8, 12, 16, 24, 36, 48, and 72 h after start of infusion; trough samples on Days 5 - 9

Table S1. Summary of clinical studies used to develop the population PK model for telavancin

Study	Phase	Study Design	Subjects/ Patients (n)[a]	Telavancin IV Dosing Regimens	Scheduled PK Sample Collection Times
TLV-2015-011 [10]	1	Open-label, single period, single-dose PK study conducted in healthy adult male and female subjects.	32	Group A (BMI 18.5 - 29.9 kg/m^2):<br>Weight 50 - 74.9 kg: 500 mg (N = 4)<br>Weight 75 - 99.9 kg: 750 mg (N = 4)<br>Group B (BMI 30 to 34.9 kg/m^2):<br>Weight 90 - 99.9 kg: 750 mg (N = 4)<br>Weight 100 - 115 kg: 1000 mg (N = 4)<br>Group C (BMI 35 to 39.9 kg/m^2):<br>Weight 105 - 130 kg: 1000 mg (N = 8)<br>Group D (BMI ≥ 40 kg/m^2):<br>Weight ≥ 120 kg: 1000 mg (N = 8)	On Day 1: just prior to infusion and at 0.5, 0.95, 1.05, 1.25, 1.5, 2, 4, 6, 8, 12, 24, and 48 h following the start of infusion
I6424-202a (FAST) [11]	2	Double blind, active controlled, parallel-group, multicenter, multinational study in patients with cSSSI	84	7.5 mg/kg q24h infused over 1 h for 4 – 14 days	On Days 3, 4 or 5: Pre-dose; just after end of infusion, and at 0.5, 1, 3, 8 (± 2 h), and 23 h after the end of infusion.
I6424-202b (FAST 2) [12]	2	Double blind, active controlled, parallel-group, multicenter, multinational study in patients with cSSSI	115	7.5 or 10 mg/kg q24h[b] for 4 – 14 days	On Days 3, 4, or 5: Pre-dose, at end of infusion, and at 0.5, 1, 3, 8 (± 2 h), and 23 h after the end of infusion.
I6424-203a [13]	2	Randomized, double-blind, parallel-group, multinational study in patients with *S. aureus* bacteremia	29	10 mg/kg q24h infused over 1 h for 14 days	On Day 4 (± 1 day): just prior to infusion, 1, 1.5, 2, 4, 9, and 24 h following the start of infusion

Table S1. Summary of clinical studies used to develop the population PK model for telavancin

Study	Phase	Study Design	Subjects/ Patients (n)[a]	Telavancin IV Dosing Regimens	Scheduled PK Sample Collection Times
I6424-0017 (ATLAS 1) [14]	3	Double-blind, active-controlled, parallel-group, multicenter, multinational study in patients with cSSSI	499	7.5 or 10 mg/kg q24h[b,c,d] for 7 – 14 days	On Day 4 (± 1 day): Pre-dose, and at 0.25 to 0.5 h, 1 to 1.5, and 2 to 3.5 h after the start of infusion
I6424-0018 (ATLAS 2) [14]	3	Double-blind, active-controlled, parallel-group, multicenter, multinational study in patients with cSSSI	522	7.5 or 10 mg/kg q24h[b,c,d] for 7 – 14 days	On Day 4 (± 1 day): Pre-dose, and at 0.25 to 0.5 h following the start of the infusion (during the infusion), 1 to 1.5, and 2 to 3.5 h after the start of infusion
I6424-0015 (ATTAIN) [15]	3	Randomized, double-blind, active-controlled, parallel-group, multicenter, multinational study in patients with HABP/VABP	372	7.5 or 10 mg/kg q24h[b,c] for 7 to 21 days	On Day 4 (± 1 day): within 0.5 h prior to the start of infusion, and at 0.75 to 1 h (during the infusion), 1.25 to 1.75 h, and 6 to 12 h after the start of infusion
I6424-0019 (ATTAIN) [15]	3	Randomized, double-blind, active-controlled, parallel-group, multicenter, multinational study in patients with HABP/VABP	377	7.5 or 10 mg/kg q24h[b,c] for 7 to 21 days	On Day 4 (± 1 day): within 0.5 h prior to the start of infusion, and at 0.75 to 1 h (during the infusion), 1.25 to 1.75 h, and 6 to 12 h after the start of infusion

Table S1. Summary of clinical studies used to develop the population PK model for telavancin

Study	Phase	Study Design	Subjects/ Patients (n)[a]	Telavancin IV Dosing Regimens	Scheduled PK Sample Collection Times
TLV-2014-020 [16]	4	Open-label, prospective, single-dose, crossover, pharmacokinetic study with two arms in otherwise healthy adults with CKD5 receiving maintenance hemodialysis	8	Arm/Period 1: 5 mg/kg single dose (based upon dry weight infused over 1 h; a 3.5 h IHD session was then begun at approximately 3 h post-dose (previous IHD session was 48 h earlier)<br>Arm/Period 2: 5 mg/kg single dose (based upon dry weight infused over 1 h immediately following an IHD session)<br>14-day washout between periods	On Day 1 of each Arm/Period:<br>just prior to infusion and at 1, 1.5, 3, 6.5, 8, 24, and 48 h following the start of infusion

Note: BAL, bronchoalveolar lavage; BMI, body mass index; CKD5, Chronic Kidney Disease Stage 5; cSSSI, complicated skin and skin structure infections; ELF, epithelial lining fluid; h, hour; HABP, hospital-acquired bacterial pneumonia; IHD, intermittent hemodialysis; kg, kilogram; mg, milligram; PK, pharmacokinetic; q24h, every 24 hours; VABP, ventilator-associated bacterial pneumonia.

a. Number of subjects refers to the number of subjects that were randomized to and treated with at least one dose of telavancin, except for Study 101a which reflects the total number of subjects that participated in the study.

b. Infusion duration was not recorded in Studies I6424-202b, -0017, -0018, -0015, and -0019 and was therefore estimated in the model.

c. Telavancin dose was adjusted to 7.5 mg/kg q24h in patients with moderate renal insufficiency (CLcr of 30 - 50 mL/min) and to 10 mg/kg every 48 hours (q48h) in patients with severe renal insufficiency on hemodialysis (CLcr < 30 mL/min).

d. In Studies I6424-0017 and -0018, only the first and last dose dates and times were recorded; all dose records in between were imputed.

References

1. Shaw JP, Seroogy J, Kaniga K, Higgins DL, Kitt M, Barriere S. Pharmacokinetics, serum inhibitory and bactericidal activity, and safety of telavancin in healthy subjects. Antimicrob Agents Chemother 2005;49(1):195-201.
2. Data on file, Cumberland Therapeutics, Inc.
3. Wong SL, Barriere SL, Kitt MM, Goldberg MR. Multiple-dose pharmacokinetics of intravenous telavancin in healthy male and female subjects. J Antimicrob Chemother 2008;62:780-783.
4. Goldberg MR, Wong SL, Shaw JP, Kitt MM, Barriere SL. Single-dose pharmacokinetics and tolerability of telavancin in elderly men and women. Pharmacother 2010:30(8):806-811.

5. Sun HK, Duchin K, Nightingale CH, Shaw JP, Seroogy J, Nicolau DP. Tissue penetration of telavancin after intravenous administration in healthy subjects. Antimicrob Agents Chemother 2006;50(2):788-790.
6. Gotfried MH, Shaw JP, Benton BM, Krause KM, Goldberg MR, Kitt MM, Barriere SL. Intrapulmonary distribution of intravenous telavancin in healthy subjects and effect of pulmonary surfactant on *in vitro* activities of telavancin and other antibiotics. Antimicrob Agents Chemother 2008;52(1):92-97.
7. Goldberg MR, Wong SL, Shaw JP, Kitt MM, Barriere SL. Lack of effect of moderate hepatic impairment on the pharmacokinetics of telavancin. Pharmacother 2010:30(1):35-42.
8. Wong SL, Goldberg MR, Ballow CH, Kitt MM, Barriere SL. Effect of telavancin on the pharmacokinetics of the cytochrome P450 3A probe substrate midazolam: a randomized, double-blind, crossover study in healthy subjects. Pharmacother 2010:30(2):136-143.
9. Wong SL, Sorgel F, Kinzig M, Goldberg MR, Kitt MM, Barriere SL. Lack of pharmacokinetic drug interactions following concomitant administration of telavancin with aztreonam or piperacillin/tazobactam in healthy participants. J Clin Pharmacol 2009;49:816-823.
10. Bunnell KL, Pai MP, Sikka M, Bleasdale SC, Wenzler E, Danziger LH, Rodvold KA. Pharmacokinetics of telavancin at fixed doses in normal-body-weight and obese (Classes I, II, and III) adult subjects. Antimicrob Agents Chemother 2018;62:e02475-17.
11. Stryjewski ME, O'Riordan WD, Lau WK, Pien FD, Dunbar LM, Vallee M, Fowler Jr VG, Chu VH, Spencer E, Barriere SL, Kitt MM, Cabell CH, Corey GR. Telavancin versus standard therapy for treatment of complicated skin and soft-tissue infections due to Gram-positive bacteria. Clin Infect Dis 2005;40:1601-1607.
12. Stryjewski ME, Chu VH, O'Riordan WD, Warren BL, Dunbar LM, Young DM, Vallee M, Fowler Jr VG, Morganroth J, Barriere SL, Kitt MM, Corey GR. Telavancin versus standard therapy for treatment of complicated skin and skin structure infections caused by Gram-positive bacteria: FAST 2 study. Antimicrob Agents Chemother 2006;50(3):862-867.
13. Stryjewski ME, Lentnek A, O'Riordan W, Pullman J, Tambyah PA, Miro JM, Fowler Jr VG, Barriere SL, Kitt MM, Corey GR. A randomized Phase 2 trial of telavancin versus standard therapy in patients with uncomplicated *Staphylococcus aureus* bacteremia: the ASSURE study. BMC Infect Dis 2014;14:289.
14. Stryjewski ME, Graham DR, Wilson SE, O'Riordan W, Young D, Lentnek A, Ross DP, Fowler VG, Hopkins A, Friedland HD, Barriere SL, Kitt MM, Corey GR. Telavancin versus vancomycin for the treatment of complicated skin and skin-structure infections caused by Gram-positive organisms. Clin Infect Dis 2008;46:1683-1693.
15. Rubinstein E, Lalani T, Corey GR, Kanafani ZA, Nannini EC, Rocha MG, Rahav G, Niederman MS, Kollef MH, Shorr AF, Lee PC, Lentnek AL, Luna CM, Fagon JY, Torres A, Kitt MM, Genter FC, Barriere SL, Friedland HD, Stryjewski ME. Telavancin versus vancomycin for hospital-acquired pneumonia due to Gram-positive pathogens. Clin Infect Dis 2011;52(1):31-40.
16. Gharibian KN, Lewis SJ, Heung M, Segal JH, Salama NN, Mueller BA. Telavancin pharmacokinetics in patients with chronic kidney disease receiving haemodialysis. J Antimicrob Chemother 2022:77:174-180.

Table S2. Summary of the number of subjects available along with the number of telavancin plasma concentrations included in and excluded from the population PK analyses

Study	Phase	Infection Type	Number of Subjects Available	Number of Telavancin Conc. Included	Number of Excluded Telavancin Conc. < LLOQ	Number of Other Telavancin Conc. Excluded
0016	1	Healthy	16	180	30	0
I6424-0032	1	Healthy	16	192	0	0
I6424-0035	1	Healthy	24	576	0	0
I6424-101a	1	Healthy	73	1236	35	46
I6424-103a	1	Healthy	29	267	14	0
I6424-104a	1	Healthy	74	749	30	13
I6424-105a	1	Healthy	16	160	0	0
I6424-107a	1	Healthy	8	64	0	0
I6424-108a	1	Healthy	20	158	0	2
9809-CL-1407	1	Healthy	45	493	0	2
9809-CL-2403	1	Healthy	43	657	116	0
9809-CL-3101	1	Healthy	24	677	65	1
TLV-2015-011	1	Healthy	13[a]	155	1	0
I6424-202a	2	cSSSI	52	316	0	6
I6424-202b	2	cSSSI	80	538	1	21
I6424-203a	2	Bacteremia	18	96	2	4
I6424-0015	3	HABP/VABP	116	450	1	20
I6424-0017	3	cSSSI	261	996	3	32
I6424-0018	3	cSSSI	164	612	1	39
I6424-0019	3	HABP/VABP	105	407	3	17
TLV-2014-020	4	Healthy	8	109	1	2
**Total used for developing the final population PK model**			**1205**	**9088**	**303**	**205**

Note: cSSSI, complicated skin and skin structure infection; HABP, hospital-acquired bacterial pneumonia; LLOQ, lower limit of quantitation; VABP, ventilator-associated bacterial pneumonia.

a. Data from only 13 out of the 32 subjects enrolled in this study were available at the time the population PK analysis was conducted.

Table S3. Summary of the most statistically significant covariate effect in each step of forward selection

Step	Parameter	Covariate	Function	OBJF	ΔOBJF	df	p-value	ω^2_{CL} %CV	ω^2_{Vc} %CV	ω^2_{CLd} %CV	ω^2_{Vp} %CV	ω^2_{D1} %CV	ω^2_{CLDL} %CV
		Base structural model		41302.616				31.7	42.6	38.5	26.0	23.1	24.7
1	Vc	Age	Power	41118.925	183.691	1	< 0.00001	31.6	38.4	38.8	25.4	23.5	24.4
2	Vc	Weight	Power	40886.310	232.615	1	< 0.00001	31.9	31.6	38.3	23.8	25.1	24.4
3	CL	Weight	Power	40727.734	158.576	1	< 0.00001	29.2	32.3	40.0	23.9	27.5	24.5
4	CL	Infection Type	Proportional Shift	40514.804	212.930	2	< 0.00001	26.4	32.7	40.2	24.5	27.3	24.7
5	Vc	Infection Type	Proportional Shift	40284.927	229.877	2	< 0.00001	26.8	28.5	40.3	24.4	28.2	25.0
6	Vp	Weight	Power	40169.998	114.929	1	< 0.00001	27.2	28.3	40.2	21.9	28.3	25.0
7	Vp	Age	Power	40093.575	76.423	1	< 0.00001	26.9	28.1	40.3	20.5	28.5	25.3
8	Vp	Infection Type	Proportional Shift	40055.357	38.218	2	< 0.00001	27.0	28.0	40.2	20.4	28.5	25.3
9	CLd	Weight	Power	40022.340	33.017	1	< 0.00001	27.0	28.1	39.7	20.3	28.6	25.0
10	Vp	BMI	Power	39997.847	24.493	1	< 0.00001	27.5	28.3	39.6	18.5	27.5	25.0
11	Vc	Sex	Proportional Shift	39989.782	8.065	1	0.00451	27.1	28.3	39.3	18.8	27.5	24.9
12	CL	Age	Power	39981.650	8.132	1	0.00435	26.7	27.8	39.5	18.6	28.0	24.8
13	CLd	Age	Power	39963.022	27.040	1	< 0.00001	27.2	28.1	36.7	18.8	28.3	23.8

Note: CL, clearance; CLd, distribution clearance between central and peripheral compartment; CL_{DL}, dialysis clearance; %CV, percent coefficient of variation; D1, infusion duration; df, degrees of freedom; OBJF, objective function; ω^2, interindividual variability; Vc, volume of distribution of the central compartment; Vp, volume of distribution of the peripheral compartment.

Table S4. ELF population PK model parameter estimates and associated standard errors

Parameter	Final estimate	%SEM	Shrinkage
k_{13} (hr^{-1})	0.0107	42.9	
k_{30} (hr^{-1})	0.154	48.2	
ω^2 for k_{30}	0.113 (33.6% CV)	62.4	21.6
Residual variability (σ^2)			
Additive component for ELF	0.316 (0.562 mg/L)	FIXED	
CCV component for ELF	0.00985 (9.92% CV)	FIXED	

Note: CCV, constant coefficient of variation; CV, coefficient of variation; ELF, epithelial lining fluid; hr, hours; k_{13}, first-order transfer from the central compartment to ELF; k_{30}, first-order elimination from the ELF compartment; L, liters; mg, milligrams; ω^2, interindividual variability; %SEM, percent standard error of the mean; σ^2, residual variability.

Figure S1. Goodness-of-fit plots for the final population PK model for telavancin

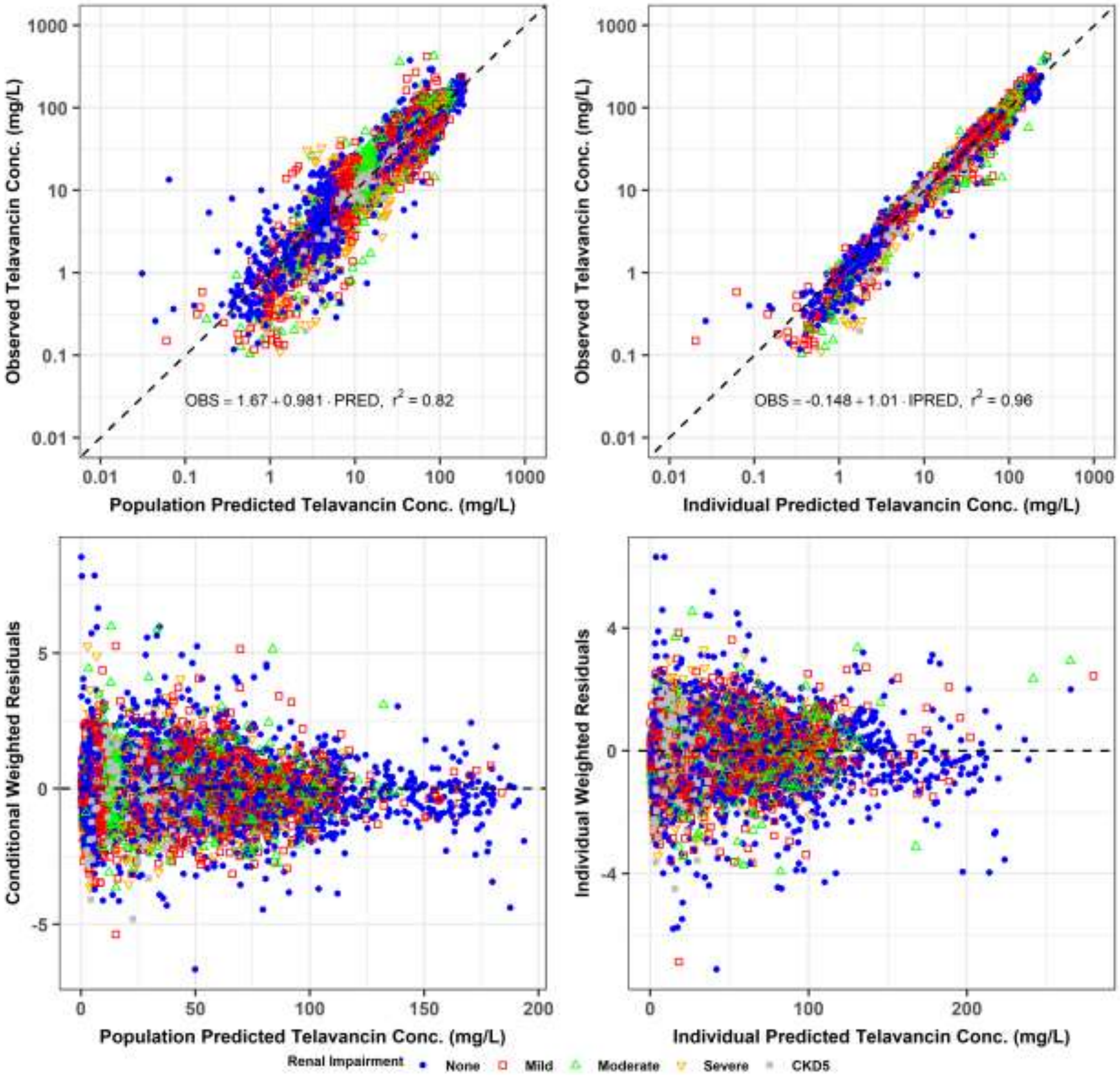

Note: CKD5, Chronic Kidney Disease Stage 5; L, liters; mg, milligrams. No renal impairment, CLcr ≥ 90 mL/min/1.73 m^2; mild impairment, CLcr ≥ 60 to < 90 mL/min/1.73 m^2; moderate impairment, CLcr ≥ 30 to < 60 mL/min/1.73 m^2; severe impairment, CLcr < 30 mL/min/1.73 m^2 and not on hemodialysis; CKD5, on hemodialysis.

Figure S2. Individual post-hoc clearance versus typical value of clearance following bootstrap simulation of 2000 patients with HABP/VABP, cSSSI or uncomplicated bacteremia

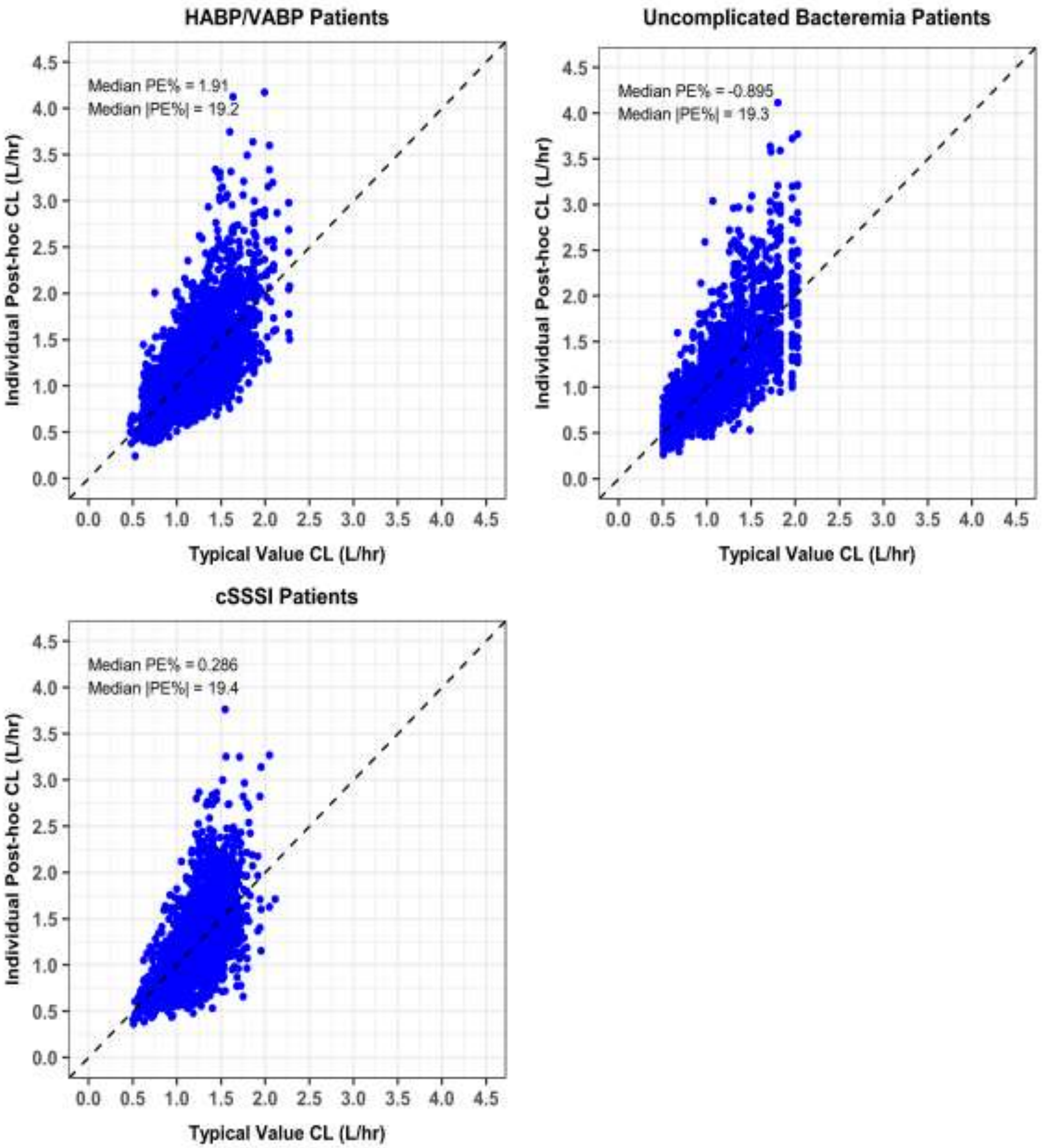

Figure S3. Goodness-of-fit plots for the sequential fit of the population PK model to the telavancin ELF data from healthy Phase 1 subjects in Study I6424-108a

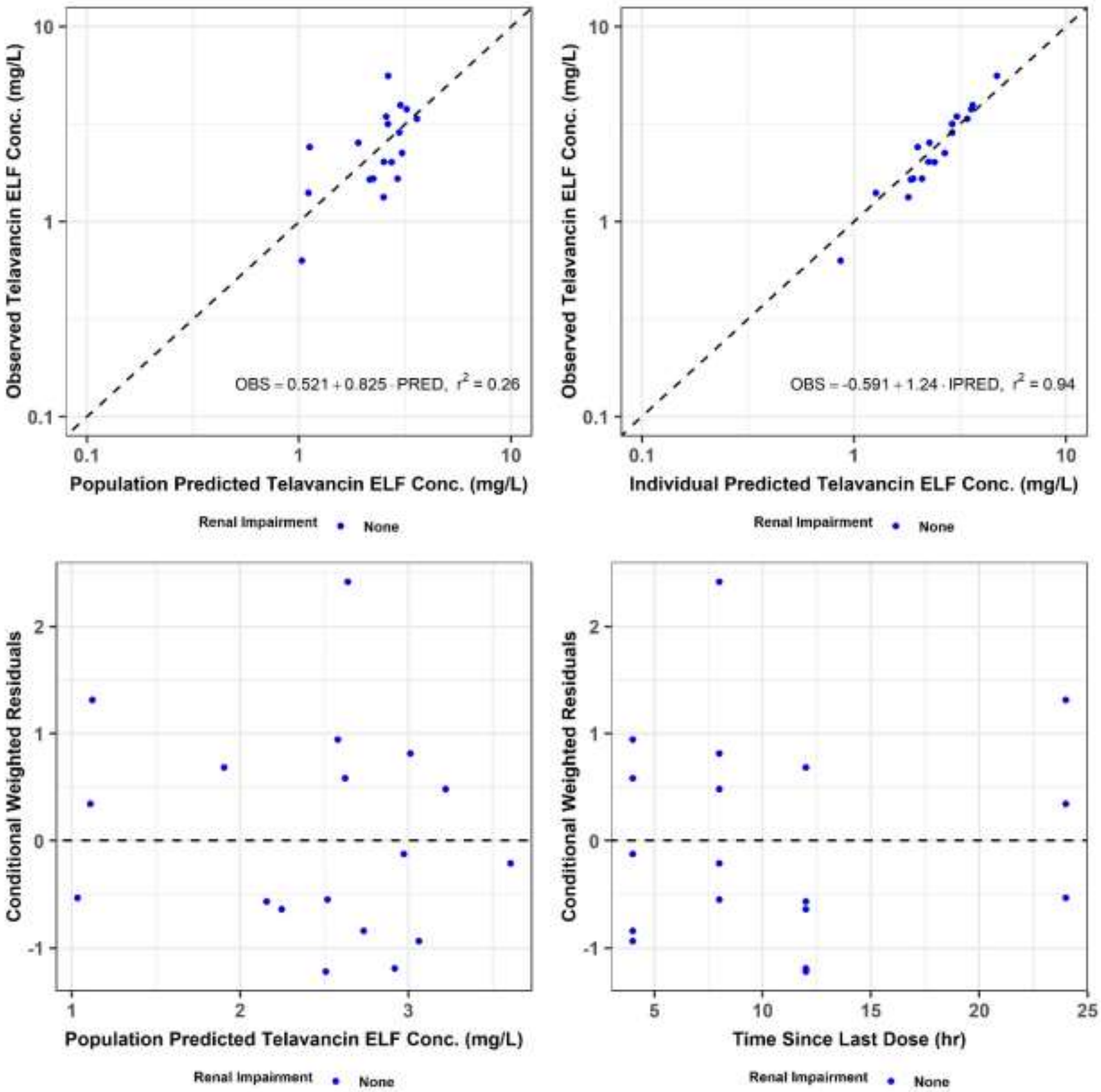

Note: Two subjects had telavancin ELF concentrations that were below the lower limit of quantitation and were thus excluded from this figure.
